# Case report: Para-testicular spindle cell lipoma suspected of well-differentiated liposarcoma

**DOI:** 10.3389/fruro.2024.1400674

**Published:** 2024-06-03

**Authors:** Kengo Fujiwara, Kengo Fujimoto, Emi Ibuki, Ryo Ishikawa, Yushi Hayashida

**Affiliations:** ^1^ Department of Urology, Sakaide City Hospital, Sakaide, Japan; ^2^ Department of Radiology, Sakaide City Hospital, Sakaide, Japan; ^3^ Department of Diagnostic Pathology, Faculty of Medicine, Kagawa University, Miki-cho, Japan

**Keywords:** case report, spindle cell lipoma, liposarcoma, scrotum, para-testicular tumor, radical inguinal orchidectomy

## Abstract

Spindle cell lipoma is a relatively rare benign tumor that can occur in the posterior neck, shoulder, and upper back. Herein, we present a case of intrascrotal spindle cell lipoma in a 71-year-old male who presented with a mass in the left scrotum that had developed over 2 years. Contrast-enhanced computed tomography (CT) revealed a 5.7cm mass accompanying enhanced solid components. Magnetic resonance imaging (MRI) showed a heterogeneous signal intensity. Therefore, a well-differentiated liposarcoma derived from the spermatic cord was suspected; therefore, the patient underwent radical inguinal orchidectomy with high ligation of the spermatic cord. Histopathological examination revealed mature adipocytes and bland-spindle cells. Immunohistochemically, the tumor cells were positive for CD-34 and negative for CDK4, MDM2, and p16. These findings indicated a spindle cell lipoma. Surgical margins were negative. Three months after surgery, no relapse was observed. This case underscores the rarity of para-testicular spindle cell lipoma. While CT and MRI play crucial roles in disease diagnosis, they may not detect all lesions. To prevent overtreatment, it’s essential to also consider pre-surgical examinations and intraoperative findings.

## 1 Introduction

Most para-testicular tumors in adults are benign, and only 3% are malignant (1). Primary para-testicular masses are rare and are reported in only 3–16% of all patients undergoing scrotal ultrasonography. Approximately 70% of para-testicular masses are located in the spermatic cord (2). The most common histological type is the liposarcoma (3). While the morbidity of spindle cell lipoma remains unclear, it is reported to be 4.9% (6/125) in cases involving the genitourinary tract or spermatic cord (4). However, morbidity rates for spindle cell lipoma in the scrotum may be even lower. Computed tomography (CT) and magnetic resonance imaging (MRI) findings are similar in both tumors (5). Herein, we report a case of a patient initially suspected to have sarcoma but later diagnosed with spindle cell lipoma. This highlights the significance of precise diagnosis, preventing unnecessary treatment.

## 2 Case description

**Figure 1:**
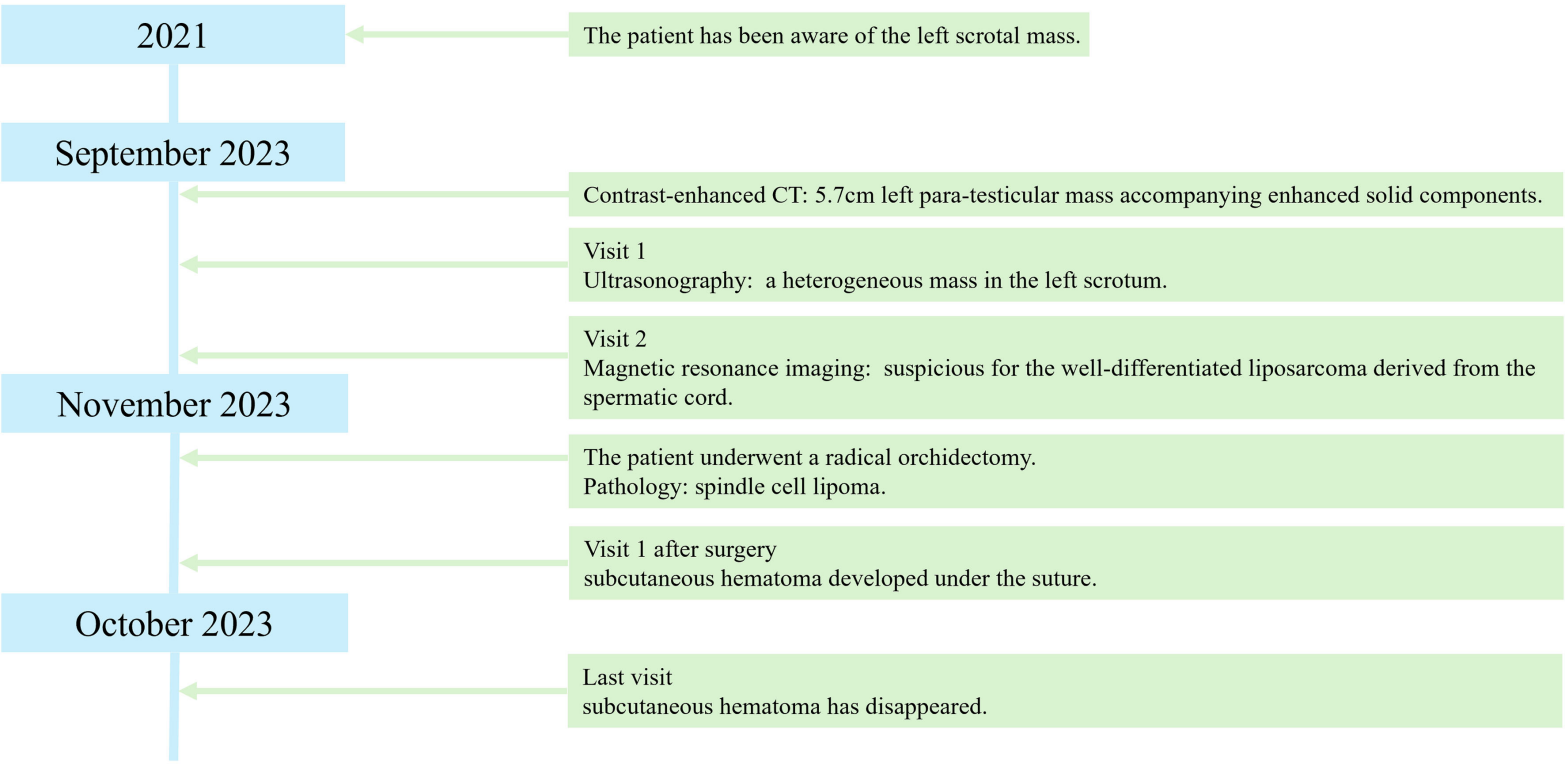
Clinical course.

**Figure 2:**
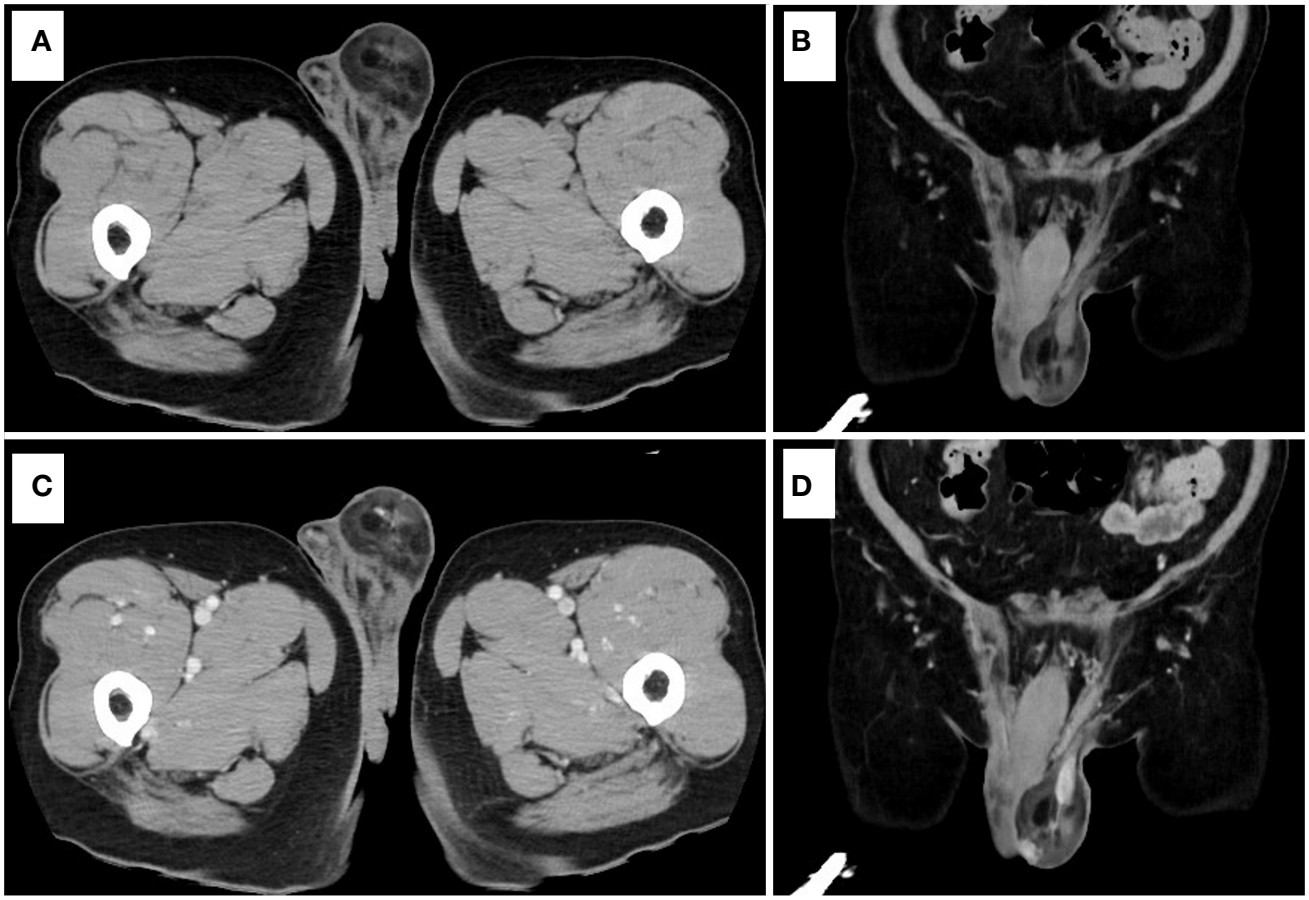
Contrast-enhanced CT. **(A)** Plain CT (axial view). **(B)** Plain CT (coronal view). **(C)** Contrast-enhanced CT (axial view). **(D)** Contrast-enhanced CT (coronal view).

**Figure 3:**
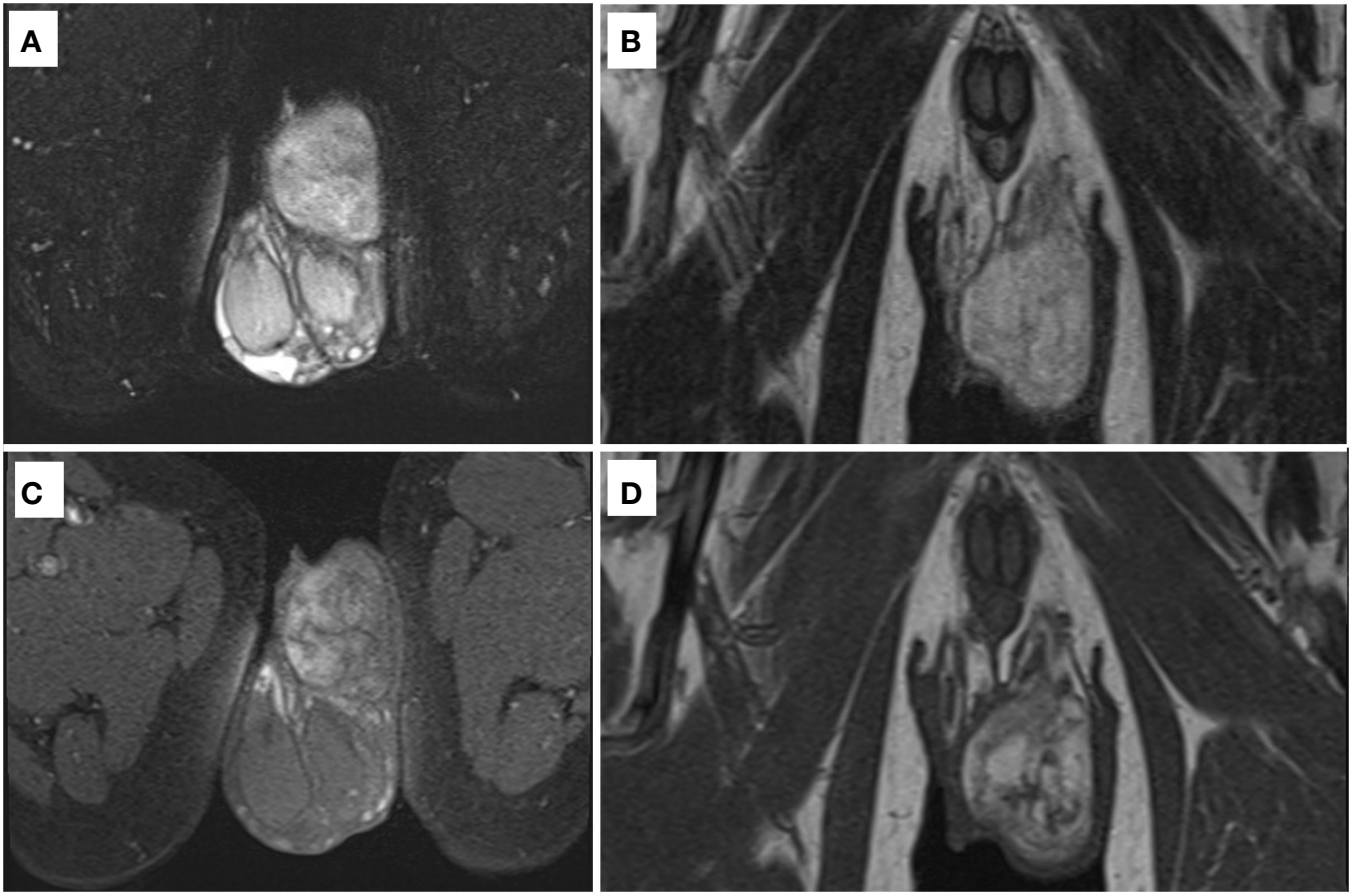
MRI. **(A)** T2-weighted image (axial view) with fat suppression. **(B)** T2-weighted image (coronal view). **(C)** T1-weighted image (axial view) with fat suppression. **(D)** T1-weighted image (coronal view).

A 71-year-old man was found to have a lesion suggestive of well-differentiated liposarcoma arising from the spermatic cord on contrast-enhanced CT (**Figure 1**). The patient was then referred to our department for further examination and treatment. The patient did not complain of pain but noticed a gradually enlarging mass over two years. There were no significant medical or family history findings. Physical examination revealed a palpable, mobile, and non-tender mass in the upper part of the left testis within the scrotum. Testicular tumor markers showed lactate dehydrogenase levels at 229 IU/L, alpha-fetoprotein levels at 2.95 ng/mL, and beta-human chorionic gonadotropin levels exceeding 1.00 ng/mL. CT revealed a 5.7 cm mass accompanying a lipoma in the left scrotum. Contrast-enhanced CT revealed enhancement of the solid components and no metastasis (**Figure 2**). In addition, scrotal ultrasonography revealed a heterogeneous mass in the left scrotum (not shown). The mass was more hyperechoic than the testis. On T1- and T2-weighted MRI, the lesion displayed a varied signal intensity (**Figure 3**). Well-differentiated liposarcomas typically exhibit specific CT features, including macroscopic fat composing at least 75% of the tumor, smooth and lobulated margins, thick septa (>3 mm), a tendency to nodularity, and mild or inconsistent low enhancement (6). In this instance, the tumor primarily comprised fatty components with a nodular aspect. Following contrast injection, mild, variable enhancement was noted. These observations initially indicated a well-differentiated liposarcoma. Considering the invasion of the subcutaneous tissue, enucleation might not have provided a complete cure. Therefore, radical inguinal orchidectomy with high ligation of the spermatic cord was performed. We preserved the external spermatic fascia and used blunt dissection to develop a plane between the mass capsule and the dermis. The tumor was situated in the para-testicular region, with both the tumor and spermatic cord encased within the external spermatic fascia. We intentionally refrained from cutting this fascia during the procedure to prevent tumor migration. Post-surgery, the tumor was effortlessly detached from the spermatic cord. The tumor was soft and smooth and appeared to arise from the fat tissue rather than from the spermatic cord.

## 3 Diagnostic assessment

**Figure 4:**
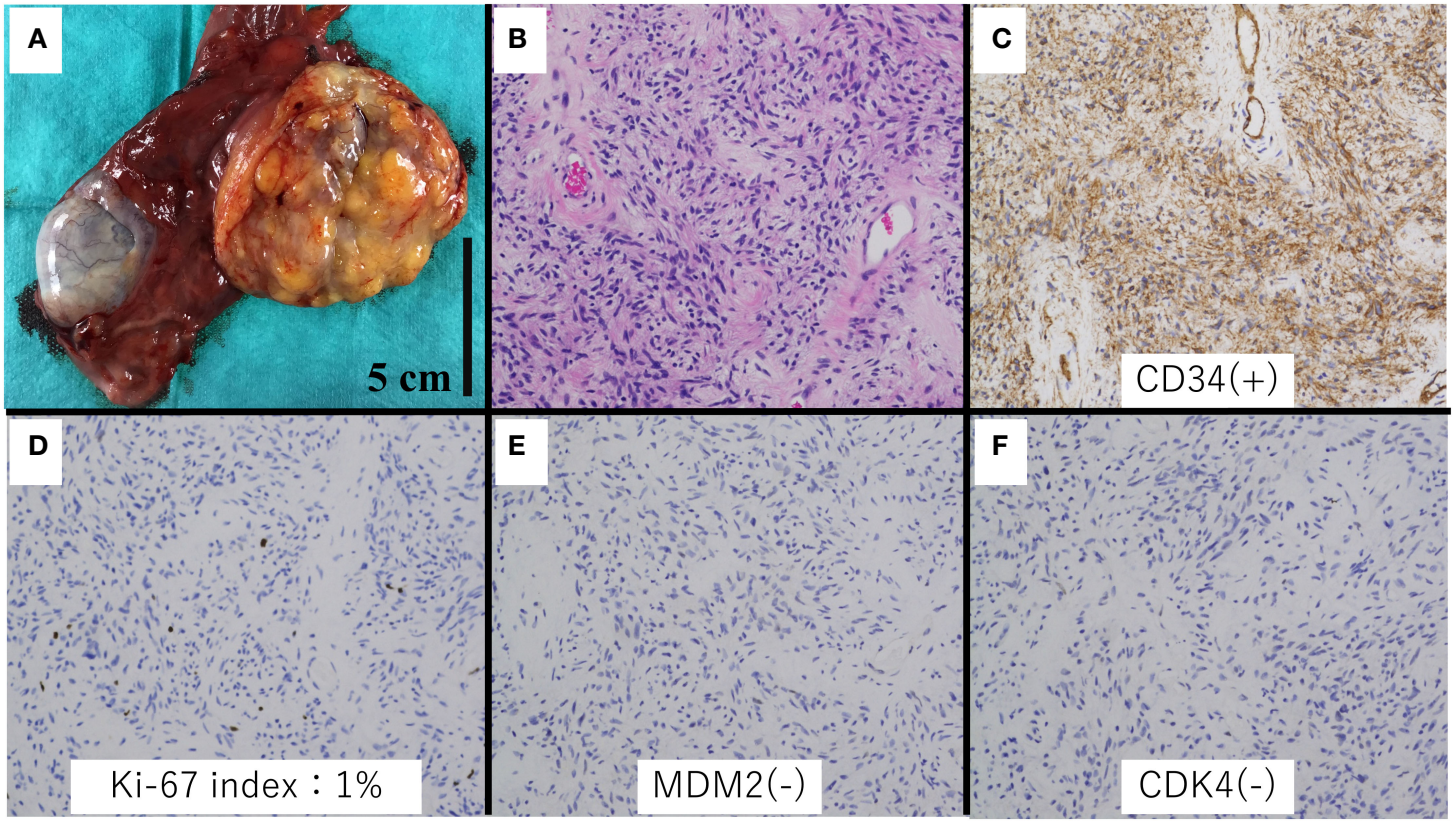
Histologic and immunohistochemical analysis of the specimen. **(A)** Gross appearance. **(B)** Spindle cell lipoma area (hematoxylin and eosin stain, ×20 magnification). **(C)** Immunohistochemical staining showed CD34 positivity in the spindle cells (×20 magnification). **(D)** Ki-67 index was 1%. **(E, F)** MDM2 and CDK4 were negative (×20 magnification).

Grossly, the tumor was a well-circumscribed mass measuring 70 × 50 × 30 mm and had a yellow or grayish-white cut surface (**Figure 3**). Histologically, the mass consisted of mature adipocytes with partially lobulated growth and bland spindle cells (**Figure 4**). Immunohistochemically, the tumor cells were positive for CD-34 and negative for CDK4, MDM2, and P16.

These findings confirmed a diagnosis of spindle cell lipoma with negative surgical margins. Despite the development of a subcutaneous hematoma beneath the suture, it resolved within a month. The patient is currently undergoing a follow-up three months post-operation, showing no signs of relapse.

## 4 Discussion

Spindle cell lipoma was first reported by Enzinger and Harvey in 1975. Spindle cell lipoma is a relatively rare benign adipocytic tumor comprising mature adipocytes and spindle cells (7). It often occurs in the subcutaneous tissues of the back, shoulders, or posterior neck. It typically presents as a relatively small (< 5 cm), mobile, slow-growing, and painless mass. It occurs mainly in males between the ages of 50–70 years (5). Although the tumor size of 5.7 cm may not align with typical spindle cell lipoma dimensions, it is noteworthy that spindle cell lipomas generally do not metastasize and are often treated with a simple incision. While scrotal spindle cell lipoma cases are rare, reported instances have been successfully managed with surgery, demonstrating no recurrence (8). In adults, over 75% of primary para-testicular tumors arise from the spermatic cord and represent 7–10% of all intrascrotal tumors. Of these, 20% are liposarcomas (9). Most para-testicular liposarcomas manifest as painless, slow-growing inguinal masses. These tumors are often misdiagnosed as hydroceles, lipomas, funicular cysts, testicular tumors, or inguinoscrotal hernia (10). It is difficult to identify the origin based on the size of the tumor and degree of adhesion (11).

Spindle cell lipoma and well-differentiated liposarcoma share characteristics of mature adipocytes and hyperechogenicity in fatty areas, often displaying heterogeneous features within solid components (5, 12). Contrast-enhanced ultrasound has been suggested as a valuable diagnostic tool, as evidenced by a meta-analysis indicating its high accuracy in detecting malignant masses, with an overall accuracy of 0.96. The sensitivity, positive predictive value, specificity, and negative predictive value of contrast-enhanced ultrasound for detecting malignancies were reported as 0.86, 0.73, 0.87, and 0.91, respectively (13).

MRI and CT revealed a heterogeneous mass with increased signals on both T1 and T2 imaging, with signal defects evident upon fat suppression. Diffusion-weighted imaging indicated restricted diffusion, and mild, variable enhancement was noted post-contrast injection. Radiographs typically reveal nonspecific soft tissue masses in liposarcoma cases. CT scans often display slightly increased attenuation in the adipose component compared to subcutaneous fat, with tissue attenuation similar to or slightly lower than that of skeletal muscle in non-adipose areas. The non-adipose component typically enhances contrast-enhanced CT. In MRI, non-adipose components exhibit low to intermediate signal intensity on T1-weighted images and intermediate to high signal intensity on T2-weighted images (5). Given these imaging findings, liposarcoma could not be ruled out. Spindle cell lipomas may contain non-adipose tissue, leading to contrast enhancement. Well-differentiated liposarcomas commonly display thick septa and soft tissue nodularity, with similar features observed in non-fatty areas.

Thus, it is difficult to distinguish between spindle cell lipomas and sarcomas by qualitatively using radiographic tools. Performing a quantitative assessment of MRI parameters may help differentiate superficial spindle cell lipomas from well-differentiated liposarcoma. Reported metrics include maximum diameter, non-fatty area on T1-weighted images, and solid hyperintense area on fat-suppressed T2-weighted images (14). Additionally, ^18^F-PSMA uptake has emerged as a potential new parameter for differentiation (15).

The origin of the tumor may provide important information. Spindle cell lipomas occur in subcutaneous tissue, while well-differentiated liposarcomas occur in deep soft tissue (16). Spindle cell lipomas typically present as solitary, mobile, slow-growing, painless subcutaneous masses. They have no metastatic potential, and there are no reports of malignant transformation. Liposarcomas usually present as large painless masses in the retroperitoneum, extremities, and para-testicular regions. However, the intrascrotal space is narrow, which makes it difficult to distinguish between the two lesions.

Spindle cell lipomas typically manifest as relatively small masses, often measuring less than 5 mm. In contrast, well-differentiated liposarcomas tend to be larger. In our case, the presentation of a 5.7 mm mass raised suspicion of well-differentiated liposarcoma due to its larger size.

Histopathology, spindle cell lipoma, and well-differentiated liposarcoma are composed of mature adipocytes. Spindel cell lipomas consist of bland spindle cells and are positive for CD34 and negative for MDM2 and CDK4. On the other hand, well-differentiated liposarcomas consist of atypical, hyperchromatic stromal cells and are negative for CD34 and positive for MDM2 and CDK4. Although CD34 positivity is characteristic of spindle cell lipomas, there have been reported cases of well-differentiated liposarcomas testing positive for CD34, as observed in a study from 2020 (17). Thus, several immunohistochemical stains may be necessary for accurate differentiation between spindle cell lipomas and well-differentiated liposarcomas.

Presurgical biopsy is not necessary for all patients. Image-guided core needle biopsy is preferred over open surgical biopsy and should be performed if neoadjuvant therapy is being considered or if a malignancy other than sarcoma is suspected (18). According to the National Comprehensive Cancer Network guidelines, both image-guided core needle biopsy and open surgical biopsy are suggested for diagnostic evaluation. The accuracy of biopsy techniques has been extensively studied, with core needle biopsy demonstrating a reported 96% accuracy in diagnosing malignancy and incisional biopsy showing a 100% accuracy (p=0.91). In a recent meta-analysis involving 17 studies and 2680 patients, sensitivity estimates for core needle biopsy and open incisional biopsy were pooled and calculated at 97% (95% confidence interval [CI], 95%-98%) and 96% (95% CI, 92–99%), respectively. The pooled specificity estimates were 99% (95% CI, 97%-99%) for core needle biopsy and 100% (95% CI, 94%-100%) for open incisional biopsy. Additionally, the complication rates after core needle biopsy and open incisional biopsy were reported as 1% and 4%, respectively (19).

Given that core needle biopsy demonstrates comparable accuracy to open incisional biopsy and lower complication rates, we have determined that image-guided core needle biopsy is the preferred option over open surgical biopsy. In this case, the patient underwent radical inguinal orchidectomy without a preoperative pathological diagnosis, resulting in the loss of the left testis. If a benign pathology had been suspected, enucleation of the mass could have been performed. According to the Japanese guidelines for soft tissue tumors, a preoperative diagnosis is preferred. However, unplanned excisions and excisions without preoperative estimation continue to be performed regularly. According to the National Comprehensive Cancer Network guidelines, an image-guided core needle biopsy should be performed if the mass is suspected to be a malignancy other than sarcoma. The accuracy of malignancy diagnosis is reportedly 96% (20). Our patient was of advanced age; therefore, he willingly underwent a radical inguinal orchidectomy. However, the risks of surgery cannot be ignored. The complications associated with radical inguinal orchidectomy include postoperative pain, phantom testis syndrome, and reduced fertility. Thus, preoperative diagnosis may help prevent overtreatment (21). Since our patient consented to undergo radical inguinal orchidectomy, we proceeded with the procedure. In cases where there is a risk of tumor spreading, surgery without a preoperative biopsy may be deemed necessary. However, such operations can be unnecessary invasions if the tumor turns out to be benign. Additionally, certain malignant tumors may require neoadjuvant chemotherapy or radiotherapy, further highlighting the importance of accurate diagnosis before surgical intervention.

## 5 Conclusion

Herein, we report a case of spindle cell lipoma in the scrotum, preoperatively suspected to be liposarcoma. While radiological images offer valuable insights, additional information is necessary for accurate disease differentiation. Hence, we suggest preoperative biopsy, specifically core-needle biopsy, as an essential step in the management of soft tissue tumors.

## Data availability statement

The original contributions presented in the study are included in the article/supplementary material. Further inquiries can be directed to the corresponding author.

## Ethics statement

Ethical approval was not required for the study involving humans in accordance with the local legislation and institutional requirements. Written informed consent to participate in this study was not required from the participants or the participants’ legal guardians/next of kin in accordance with the national legislation and the institutional requirements. Written informed consent was obtained from the individual(s) for the publication of any potentially identifiable images or data included in this article.

## Author contributions

KFujiw: Writing – original draft, Writing – review & editing. KFujim: Conceptualization, Visualization, Writing – review & editing. EI: Conceptualization, Visualization, Writing – review & editing. RI: Conceptualization, Visualization, Writing – review & editing. YH: Conceptualization, Resources, Supervision, Writing – review & editing.
